# Identification of novel therapeutic targets for heart failure and its subtypes: Insights from proteomic and transcriptomic Mendelian randomization

**DOI:** 10.1016/j.gendis.2026.102229

**Published:** 2026-05-09

**Authors:** Haozhang Huang, Huangtao Ruan, Jin Liu, Yong Liu

**Affiliations:** a Department of Cardiology, Guangdong Provincial People's Hospital (Guangdong Academy of Medical Sciences), Southern Medical University, Guangzhou, Guangdong 510080, China; b Department of Guangdong Provincial Key Laboratory of Coronary Heart Disease Prevention, Guangdong Cardiovascular Institute, Guangdong Provincial People's Hospital, Guangdong Academy of Medical Sciences, Guangzhou, Guangdong 510080, China

Heart failure (HF) is a complex and heterogeneous clinical syndrome characterized by structural and/or functional myocardial impairment, resulting in symptoms such as dyspnea, fatigue, and fluid retention. HF represents a major global health burden, with diverse etiologies and clinical manifestations that are typically classified according to left ventricular ejection fraction (e.g., heart failure with reduced ejection fraction (HFrEF) and heart failure with preserved ejection fraction (HFpEF). Recent large-scale genome-wide association studies (GWAS) have begun to elucidate the genetic architecture underlying HF and its subtypes.^1^ Identifying novel, causally relevant protein targets is essential for advancing subtype-specific therapeutic strategies.

To elucidate causal relationships between circulating proteins and HF risk, we performed a proteome-wide Mendelian randomization (MR) analysis. Discovery GWAS summary statistics were obtained from the HERMES Consortium 2.0, encompassing 1,708,342 European participants with 139,533 all-cause HF cases and 1,568,809 controls (supplementary methods), and further refined by subtype: non-ischemic HF (42,081 cases), non-ischemic HFrEF (4975 cases), non-ischemic HFpEF (3590 cases), hypertrophic cardiomyopathy (HCM; 5900 cases, 68,359 controls), and dilated cardiomyopathy (DCM; 9365 cases, 946,368 controls). Replication GWAS data for all-cause HF (37,653 cases, 462,695 controls) and additional GWAS for ischemic HF (IHF; 17,532 cases, 443,698 controls) were obtained from the FinnGen R12 cohort.^2^ The plasma proteome data included 2923 proteins measured in 54,219 European participants via the Olink platform from the UKB-PPP study. We defined cis-pQTLs as single nucleotide polymorphisms (SNPs) within 1 Mb of the coding gene for each protein, and conducted colocalization analyses to ensure that associations were driven by shared causal variants rather than linkage disequilibrium. The MR analysis was conducted with the R package TwoSampleMR v.0.5.6. In the case of proteins with only one SNP, we applied the Wald ratio method. In the case of proteins with two or more SNPs, we applied the inverse-variance weighted method. Based on repeated analyses, a *p* value of less than 0.05 was considered nominally significant, and a false discovery rate (FDR) of less than 0.05 was considered statistically significant. For the discovery cohort, we required statistical significance, whereas for the replication cohort, nominal significance was sufficient, provided that the direction of the protein effect was consistent.

Complementary transcriptome-wide MR analyses using Summary-data-based Mendelian randomization (SMR) were performed using eQTLGen and GTEx v8 datasets across multiple tissues—including blood (*n* = 31,684), whole blood (*n* = 558), adipose subcutaneous (*n* = 479), adipose visceral omentum (*n* = 393), artery aorta (*n* = 329), artery coronary (*n* = 175), artery tibial (*n* = 476), heart atrial appendage (*n* = 316), and heart left ventricle (*n* = 327)—to assess the association between the expression level of a gene and all-cause HF (supplementary methods). *p* < 0.05/9 and p-HEIDI < 0.01 were set as thresholds for statistical significance. Additionally, we explored the effects of the selected proteins on different HF subtypes. *p* < 0.05/6 was set as the threshold for significance.

Among the 24 plasma proteins initially associated with all-cause HF in HERMES 2.0, six were successfully replicated in FinnGen R12 (Fig. 1). Notably, three proteins—ACRBP (acrosin binding protein), HYOU1 (hypoxia up-regulated protein 1), and LMOD1 (leiomodin 1)—exhibited colocalization evidence (PPH4 > 0.5). LMOD1 demonstrated consistent risk effects on all-cause HF in specific tissues, such as the adipose subcutaneous (β-SMR = −0.099, p-SMR = 6.49E-05, p-HEIDI = 0.082), adipose visceral omentum (β-SMR = −0.093, p-SMR = 1.65E-04, p-HEIDI = 0.031), and heart atrial appendage (β-SMR = −0.010, p-SMR = 5.34E-04, p-HEIDI = NA). ACRBP was robustly associated with ischemic heart failure (IHF) (odds ratio [OR] = 1.28, 95% confidence interval [CI] = 1.09–1.51, *p* = 0.002) and non-ischemic HFrEF (OR = 1.21, 95% CI = 1.09–1.34, *p* = 3.04E-04). A nominal association was also observed for non-ischemic HF (OR = 1.44, 95% CI = 1.06–1.95, *p* = 0.019), which did not survive multiple-testing correction and should therefore be interpreted with caution. HYOU1 showed a significant association with IHF (OR = 1.28, 95% CI = 1.12–1.47, *p* = 4.46E-04). In addition, suggestive associations were observed for non-ischemic HFpEF (OR = 1.36, 95% CI = 1.04–1.77, *p* = 0.026) and HCM (OR = 1.30, 95% CI = 1.03–1.64, *p* = 0.027), although these did not meet the threshold for multiple-testing correction. LMOD1 exhibited a modest protective association with non-ischemic HF (OR = 0.92, 95% CI = 0.84–1.00, *p* = 0.041), suggesting potential subtype-specific mechanisms, albeit with a relatively small effect size.

**Figure 1:**
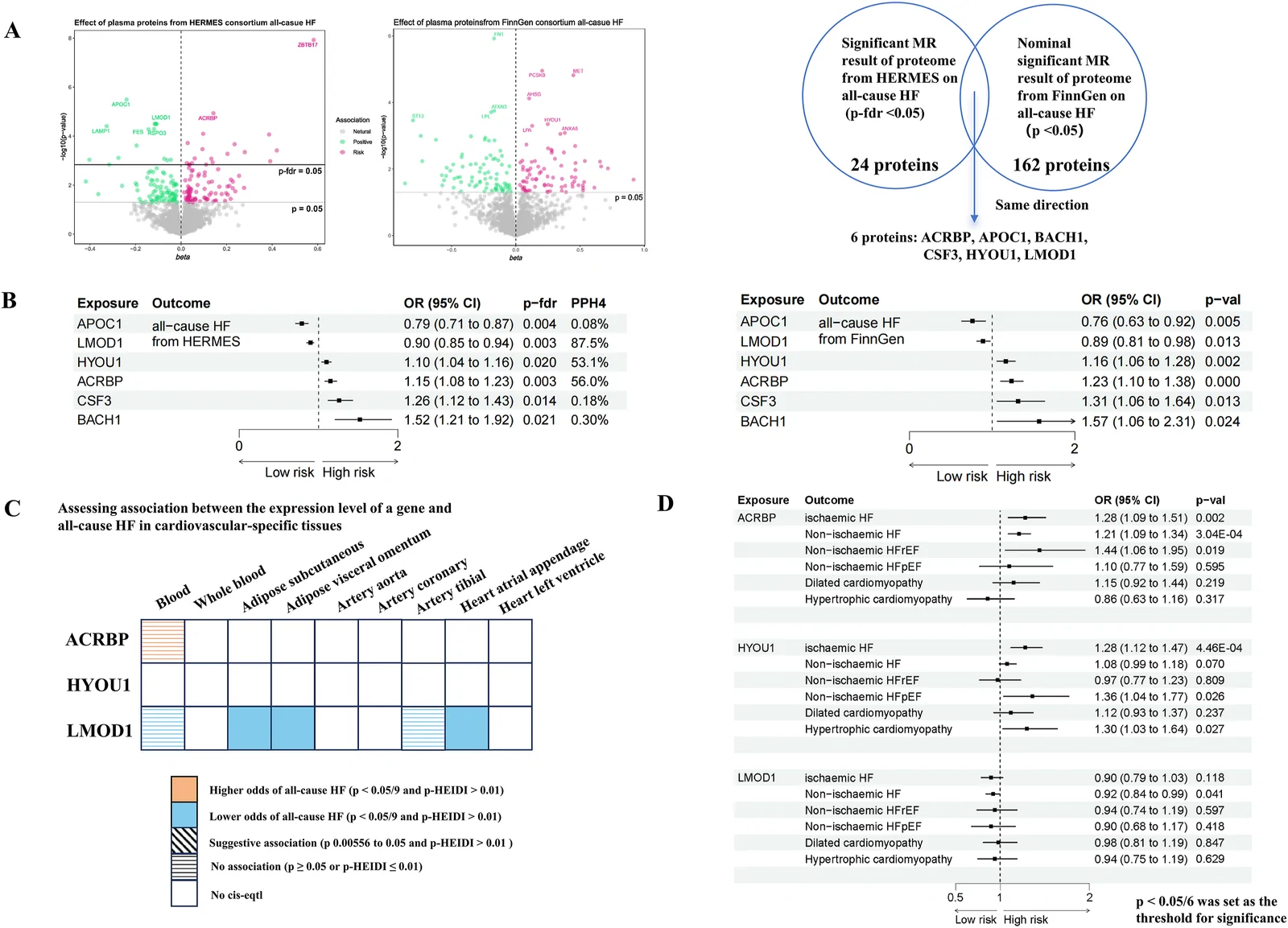
Identification of novel therapeutic targets for heart failure and its subtypes. **(A)** Volcano plot of individual proteins causally associated with all-cause HF outcome across two data sources; significant proteins are shown (*q* < 0.05) in discovery MR analysis, and nominally significant proteins (*p* < 0.05) in replication MR analysis are shown. Overlapping proteins across the two datasets are indicated. **(B)** Forest plot of identified proteins for the risk of all-cause HF and colocalization analyses. The ORs and 95% CIs of the overlapping proteins in two datasets are indicated. **(C)** Heatmap of identified protein-coding genes associated with all-cause HF risk. The color represents the β estimators from SMR analysis, where blue represents a decreased all-cause HF risk and red represents an increased all-cause HF risk per SD increase in gene expression. *p* < 0.05/6 was set as the threshold for significance. p-HEIDI ≤ 0.01 was used as an indication of heterogeneity and the possibility of influence from a nearby gene. Slashed patterns: indicate suggestive associations (*p* between 0.00556 and 0.05 and p-HEIDI > 0.01). Horizontal lines: represent no significant association (*p* ≥ 0.05 or p-HEIDI ≤ 0.01). Solid color blocks represent significant associations, where blue and red denote the direction of effect. **(D)** Forest plot of identified proteins for the risk of different HF subtypes. HF, heart failure; MR, Mendelian randomization; OR, odds ratio; CI, confidence interval; SMR, Summary-data-based Mendelian randomization; SD, standard deviation; q, false discovery rate-adjusted *p* value; p-HEIDI, HEIDI test *p* value.

LMOD1 is suggested to be involved in smooth muscle cell contractile activity and actin cytoskeleton homeostasis. Previous studies have indicated its association with cardiac conduction, and its potential role in HF may be linked to abnormalities in these processes.^3^ In our transcriptomic analysis, we found that LMOD1 has potential heart-specific effects. These findings suggest that LMOD1 may play a significant role in HF, but further protein-level validation in cardiac tissue or through mechanistic studies is necessary to better understand its role in HF pathogenesis. ACRBP, although primarily recognized as a proacrosin-binding protein, has not been previously characterized in cardiovascular diseases.^4^ However, many binding proteins with modulatory effects on extracellular matrix remodeling and cardiomyocyte function play critical roles in heart failure pathogenesis. As a hypoxia-responsive endoplasmic reticulum (ER) chaperone, HYOU1 regulates protein folding, ER-associated degradation, and apoptosis. Overexpression of HYOU1, particularly in HFpEF and HCM, may exacerbate ER stress, leading to cardiomyocyte death and fibrosis.^5^ These findings suggest that HYOU1 could play a key role in metabolic dysregulation and cardiac remodeling in these HF subtypes.

Our study is based solely on association analyses, serving as an initial exploratory investigation. It is important to note that the conclusions drawn from this study apply primarily to European populations. Therefore, functional validation of these targets, both *in vitro* and *in vivo*, is crucial to elucidate how modifying their expression or function could help mitigate the progression of HF. The statistical power in the analyses of certain HF subtypes was generally low due to small sample sizes. In future research, it would be ideal to focus on subtype-specific MR analyses using larger, more homogeneous sample sizes. Integrating data from cardiac tissue proteomics and other disease-specific biomarkers would allow for a more nuanced understanding of how proteins contribute to specific HF subtypes and their potential for targeted treatment. Additionally, large-scale, multiethnic cohort studies will be essential to refine these findings and contribute to the development of precision medicine for diverse HF subtypes. Further exploration of the impact of non-genetic factors, such as age, gender, and socioeconomic status, on the associations observed is also necessary to provide a more comprehensive understanding of the pathogenesis of HF.

In conclusion, our integrative proteome- and transcriptome-wide MR analyses emphasize the heterogeneous etiology of HF and identify multiple novel causal candidates. ACRBP, HYOU1, and LMOD1 were potentially associated with all-cause HF. Notably, ACRBP was linked to IHF and non-ischemic HFrEF, while HYOU1 was associated with IHF, non-ischemic HFpEF, and HCM.

## CRediT authorship contribution statement

**Haozhang Huang:** Writing – review & editing, Writing – original draft, Supervision, Software, Resources, Data curation. **Huangtao Ruan:** Data curation. **Jin Liu:** Writing – review & editing. **Yong Liu:** Writing – review & editing, Writing – original draft, Supervision, Conceptualization.

## Data availability

GWAS summary statistics from public databases (https://www.synapse.org/#!Synapse:syn51364943/, https://cvd.hugeamp.org/downloads.html and https://www.finngen.fi/en/access_results).

## Funding

This study was supported by the Medical Science and Technology Research Foundation of Guangdong Province (China) (No. A2022241) and the National Natural Science Foundation of China (No. 82270339, 82570393).

## Conflict of interests

The authors declare no competing interests.
