# Supplementary material for: Identification of novel therapeutic targets for heart failure and its subtypes: Insights from proteomic and transcriptomic Mendelian randomization
Source: Genes Dis. 2026 May 9;14(3):102229. doi: 10.1016/j.gendis.2026.102229 (PMC13742271; doi:10.1016/j.gendis.2026.102229)
Supplement: Supplementary file 1 [file mmc1.docx]

**Materials and Methods**

**Data sources for plasma proteins and transcriptome**

This study identified a total of 2,923 proteins within a cohort of 54,219 participants utilizing the Olink platform within the UKB-PPP. During the genetic association analysis, the models incorporated the following covariates: age, age squared, gender, age × gender interaction, age squared × gender interaction, batch, UKB center, UKB genotyping array, the time between sampling and measurement, and the first 20 genetic principal components.

Gene expression data were obtained from the eQTLGen consortium and 49 tissues sourced from the GTEx (v8) project, focusing on SNPs linked to expression changes of plasma protein-targeted genes. We focused on blood, adipose, vessels and heart tissues. Our analysis concentrated on cis-eQTLs to affirm that genetic variants were relevant to alterations in gene expression, enabling us to explore tissue-specific associations and evaluate potential off-target effects of drugs targeting these genes ^1^.

**Data sources for HF**

Summary-level data for heart failure were collected from a large-scale GWAS meta-analysis involving European ancestry participants, incorporating 42 cohorts (HERMES Consortium 2.0), 139,533 HF cases and 1,568,809 controls.^2^ Each study defined up to four HF phenotypes, which are as follows: (1) overall HF (HF_all_), (2) ni-HF, (3) ni-HFrEF and (4) ni-HFpEF. The HF_all_ phenotype includes any diagnosis of HF based on a physician’s adjudication, hospital record review or diagnosis codes. ni-HF was defined by excluding antecedent ischemic, valvular and congenital heart diseases. ni-HFrEF was defined as ni-HF with LVEF < 50% based on cardiac imaging or diagnosis of LVSD at any point. ni-HFpEF was defined as ni-HF with LVEF ≥ 50% based on cardiac imaging without a record of LVSD at any point. Study-level GWAS adjustments were made for covariates such as sex, age at baseline, principal components, and genotyping array.

**Statistical analysis**

**Proteome-wide MR investigation**

In this comprehensive proteome-wide MR analysis concentrating on drug targets, we opted to leverage cis-pQTLs as instrumental variables by applying specific selection criteria. These criteria are outlined as follows: (1) SNPs situated within ±1 Mb range of the gene locus (cis-acting pQTLs) ; (2) a stringent genome-wide significance criterion set at P<5×10^-8^ to effectively identify SNPs that demonstrate a strong association with plasma proteins; (3) to ensure the inclusion of independent SNPs and reduce the influence of linkage disequilibrium (LD) on our findings, we established a linkage disequilibrium threshold (r²) of 0.001 alongside a genetic distance cap of 10,000 kb; (4) an F-statistic greater than 10, MAF> 0.001 and INFO > 0.7 to mitigate potential bias stemming from weak instrumental variables (Figure 1).^3^ As a result, we identified 6,194 cis-pQTLs associated with 1,984 proteins, with 741 proteins having only one cis-pQTL.

**Summary-data-based MR**

We performed Summary-data-based Mendelian randomization (SMR) analysis to pinpoint potential risk genes. Utilizing GWAS and eQTL data, we assessed the relationships between the top SNPs and gene expression levels. For the eQTL expression data, we employed genomic expression datasets from the GTEx V8 project for kidney cortex samples and utilized the eQTLgen consortium data for whole blood analysis. As part of our standard procedure, we excluded SNPs exhibiting strong linkage disequilibrium, categorized by an r² value greater than 0.9, and top-associated cis-eQTLs if the MAF exceeded 0.01. Expression probes with an eQTL P-value of ≤ 5 × 10⁻⁸ were selected as the most significantly associated variants. Furthermore, we performed the heterogeneity in dependent instruments (HEIDI) test to investigate the presence of linkage within the observed associations. A P_HEIDI_ value less than 0.01 suggests that the identified association may arise from two separate genetic variants exhibiting high linkage disequilibrium^4;5^.

**Colocalization analysis**

Through the identification of common causal genetic variants associated with both the exposure and outcome, colocalization analysis further substantiates the findings derived from MR. For the proteins that exhibited positive MR outcomes, we performed colocalization analysis by SNPs located within a ±1MB window upstream and downstream of the genes related to these proteins (cis-pQTLs). We then analyzed the colocalization of these SNPs with proteins.^6^ In our colocalization assessment, PPH4 values were interpreted as follows: a probability of H4 (PPH4) of 30-50% was considered evidence of general colocalization, 50-75% indicated moderate strength of colocalization, and values exceeding 75% represented strong evidence of colocalization.

**References**

1.	Võsa U, Claringbould A, Westra HJ, et al. Large-scale cis- and trans-eQTL analyses identify thousands of genetic loci and polygenic scores that regulate blood gene expression. *Nat Genet.* 2021;53(9):1300-1310.

2.	Zheng SL, Henry A, Cannie D, et al. Genome-wide association analysis provides insights into the molecular etiology of dilated cardiomyopathy. *Nat Genet.* 2024;56(12):2646-2658.

3.	Schmidt AF, Finan C, Gordillo-Marañón M, et al. Genetic drug target validation using Mendelian randomisation. *Nat Commun.* 2020;11(1):3255.

4.	Wu Y, Zeng J, Zhang F, et al. Integrative analysis of omics summary data reveals putative mechanisms underlying complex traits. *Nat Commun.* 2018;9(1):918.

5.	Zhu Z, Zhang F, Hu H, et al. Integration of summary data from GWAS and eQTL studies predicts complex trait gene targets. *Nat Genet.* 2016;48(5):481-487.

6.	Giambartolomei C, Vukcevic D, Schadt EE, et al. Bayesian test for colocalisation between pairs of genetic association studies using summary statistics. *PLoS Genet.* 2014;10(5):e1004383.
